# Steroid versus placebo injections and wrist splints in patients with carpal tunnel syndrome: a systematic review and network meta-analysis

**DOI:** 10.1177/17531934241240380

**Published:** 2024-03-28

**Authors:** Ebubechi Adindu, Sina Ramtin, Ali Azarpey, David Ring, Teun Teunis

**Affiliations:** 1 Baylor College of Medicine, Houston, TX, USA; 2 Department of Surgery and Perioperative Care, Dell Medical School, The University of Texas at Austin, TX, USA; 3 Department of Plastic Surgery, University of Pittsburgh Medical Center, Pittsburgh, PA, USA

**Keywords:** Carpal tunnel syndrome, corticosteroids, idiopathic median neuropathy at the carpal tunnel, placebo, splint

## Abstract

A network meta-analysis of randomized controlled trials compared the effectiveness of corticosteroid injections with placebo injections and wrist splints for carpal tunnel syndrome, focusing on symptom relief and median nerve conduction velocity. Within 3 months of the corticosteroid injection, there was a modest statistically significant difference in symptom relief compared to placebo injections and wrist splints, as measured by the Symptom Severity Subscore of the Boston Carpal Tunnel Questionnaire; however, this did not meet the minimum clinically important difference. Pain reduction with corticosteroids was slightly better than with wrist splints, but it also failed to reach clinical significance. Electrodiagnostic assessments showed transient changes in distal motor and sensory latencies in favour of corticosteroids at 3 months, but these changes were not evident at 6 months. The best current evidence suggests that corticosteroid injections provide minimal transient improvement in nerve conduction and symptomatology compared with placebo or wrist splints.

## Introduction

Carpal tunnel syndrome (CTS) is the collection of symptoms and signs characteristic of idiopathic median neuropathy at the carpal tunnel. The American Academy of Orthopaedic Surgery (AAOS) treatment guidelines concluded that there is strong evidence that steroid (methylprednisolone) injections alleviate symptoms of CTS (improves patient-reported outcomes) based on a single placebo-controlled randomized controlled trial (RCT) (Atroshi et al., 2013; Graham et al., 2016), which may not be sufficient to establish conclusive results. It remains uncertain whether corticosteroid injections are palliative (relieving symptoms) or disease-modifying (preventing sensory loss and thenar atrophy) (Ioannidis, 2005). To be considered palliative, corticosteroid injections must consistently outperform non-specific factors, such as regression to the mean and placebo effects, and outperform non-invasive treatments, such as splinting. To be considered disease-modifying, corticosteroid injections should result in sustained reduction in electrodiagnostic measures of median neuropathy.

This network meta-analysis compared the relative effects of corticosteroid injections, placebo injections and splint wear on symptom intensity and median neuropathy measured by nerve conduction studies in patients diagnosed with CTS. The primary null hypothesis was that there would be no difference in symptom intensity after corticosteroid injection, placebo injection and splinting for CTS. The secondary hypothesis was that there would be no difference in nerve conduction study measures of median neuropathy between treatment modalities.

## Methods

### Search strategy and selection criteria

We followed the PRISMA guidelines, although our review was not registered (Hutton et al., 2015). We searched the PubMed, Embase and Cochrane libraries for RCTs between 1980 and 11 November 2022 using the following search criteria: (1) adult patients diagnosed with idiopathic CTS; (2) comparison of at least two types of the following treatments: corticosteroid injections, placebo injections (i.e. dextrose, saline, etc.) or wrist splints; and (3) inclusion of any patient-reported outcomes measuring symptom intensity or level of disability, and/or any electrodiagnostic measures. Studies were excluded if other medications were used in conjunction with the treatments of interest. The search was restricted to articles in the English language (Appendix A).

Three authors independently assessed all article titles and abstracts and the full texts of studies that potentially met the inclusion criteria. They abstracted the study characteristics and outcomes of interest from the included studies.

### Quality assessment

Using the Cochrane Collaboration’s risk of bias tool, two authors independently assessed the methodological quality of the included studies (Higgins et al., 2011). Differences were mediated by a third independent author. Publication bias was assessed using funnel plots.

### Study characteristics and risk of bias

We included 10 studies involving 776 patients and 819 hands. In total, 395 patients received corticosteroid injection, 179 patients received a placebo injection and 202 patients received a splint. The majority of the patients in all included studies were women, and the mean age was 52 years (Table 1).

**Table 1.** Study characteristics.

Study	Participants per modality			Mean age per modality (years)			Sex (% F) per modality			Follow-up time	Outcomes
	Steroid injection	Placebo	Splint	Steroid injection	Placebo	Splint	Steroid injection	Placebo	Splint		
De Moraes et al. (2021)	52	0	48	54.2	N/A	54.4	42/52	N/A	43/48	1, 3 and 6 months	FSS, SSS
Chesterton et al. (2018)	116	0	118	52.6	N/A	52.2	73/116	N/A	81/118	<3 months, 3–6 months, 6 months to 1 year, >1 year	FSS, SSS, VAS
So et al. (2018)	25	0	25	57.3	N/A	57.3	21/25	N/A	22/25	4 weeks	FSS, SSS
Celiker et al. (2002)	12	0	11	46.9	N/A	49.6	12/12	N/A	10/11	8 weeks	VAS, DML, DSL
Wu et al. (2018)	27	27	0	54.3	58.6	N/A	21/27	22/27	N/A	1, 3, 4 and 6 months	FSS, SSS, VAS, DML, SNCV
Karadaş et al. (2012)	20	19	0	46.4	48.4	N/A	17/20	17/19	N/A	2 and 6 months	FSS, SSS, VAS, DML, SNCV
Peters-Veluthamaningal et al. (2010)	36	33	0	56.5	57.6	N/A	27/36	26/33	N/A	2 weeks	FSS, SSS
Babaei-Ghazani et al. (2022)	27	27	0	49.3	48.6	N/A	Not reported	Not reported	N/A	2 weeks, 6 weeks, 3 months, 6 months	FSS, SSS, VAS, DML, DSL, SNCV
Armstrong et al. (2004)	43	38	0	51.9	51.2	N/A	35/43	28/38	N/A	2 weeks	FSS, SSS, DML, DSL
Atroshi et al. (2013)	37	35	0	44	49	N/A	27/37	28/35	N/A	5 weeks, 10 weeks, 6 months, 1 year	SSS

DML: distal motor latency; DSL: distal sensory latency; FSS: functional status scale; N/A: not applicable; SNCV: sensory nerve conduction velocity; SSS: symptom severity scale; VAS: visual analogue scale

All included studies were at low risk for bias in random sequence generation, outcome measurement and selective reporting. Eight of the studies had some concerns for bias due to deviations from intended interventions in light of inadequate blinding of personnel or participants (Babaei-Ghazani et al., 2022; Celiker et al., 2002; Chesterton et al., 2018; de Moraes et al., 2021; Karadaş et al., 2012; Peters-Veluthamaningal et al., 2010; So et al., 2018; Wu et al., 2018). One of the studies was at high risk for attrition bias (incomplete outcome data) (Supplementary Figure S1) (Karadaş et al., 2012; Sterne et al., 2019). The funnel plots did not indicate obvious publication bias (Appendix B).

### Outcome measures

Most studies reported outcomes at less than 3 months and at 6 months after treatment initiation. Therefore, we analysed outcomes at those time points. The functional status (FSS) and symptom severity (SSS) subscores of the Boston Carpal Tunnel Questionnaire (BCTQ) and the visual analogue scale (VAS) for pain were the most commonly used patient-reported outcome measures, so we conducted a meta-analysis of all studies reporting at least one of these metrics (Armstrong et al., 2004; Atroshi et al., 2013; Babaei-Ghazani et al., 2022; Celiker et al., 2002; Chesterton et al., 2018; de Moraes et al., 2021; Karadaş et al., 2012; Peters-Veluthamaningal et al., 2010; So et al., 2018; Wu et al., 2018).

Eight studies measured the impact of treatment on the FSS while all of the studies measured the change in SSS (Armstrong et al., 2004; Atroshi et al., 2013; Babaei-Ghazani et al., 2022; Celiker et al., 2002; Chesterton et al., 2018; de Moraes et al., 2021; Karadaş et al., 2012; Peters-Veluthamaningal et al., 2010; So et al., 2018; Wu et al., 2018). The FSS consists of eight items and measures capability; the SSS consists of 11 items and measures symptom intensity. All items are rated on a scale of 1–5. The scores were averaged among all the study participants, with a greater score indicating greater incapability.

A previous study reported a relative minimally clinically important difference (MCID) of 0.48 multiplied by the mean baseline score for SSS and 0.28 for the FSS (De Kleermaeker et al., 2019; Levine et al., 1993). The MCID signifies the minimum difference perceived as worthwhile by patients. Therefore, for clinical relevance, differences in outcomes between corticosteroid injection, placebo or splinting must exceed the MCID. In addition, MCID varies per diagnosis, treatment and time of measurement (Revicki et al., 2008).

Five studies used a VAS (0 to 10) to measure pain intensity, with higher scores indicating greater pain intensity (Babaei-Ghazani et al., 2022; Celiker et al., 2002; Chesterton et al., 2018; Karadaş et al., 2012; Wu et al., 2018). Previous studies found an MCID of 1.4 for a pain VAS (Hoogendam et al., 2022).

Five studies measured distal motor latency (normal <4.0 ms), three of which also measured the distal sensory latency (normal <3.5 ms) and three also measuring the sensory nerve conduction velocity (normal = 50–70 m/s) (Armstrong et al., 2004; Babaei-Ghazani et al., 2022; Celiker et al., 2002; Karadaş et al., 2012; Wu et al., 2018). The MCID values for these three nerve conduction study measurements are currently unknown.

### Data analysis

To assess treatment effects on outcomes, we calculated the mean score change from baseline for each group. For studies that did not report this, we computed these values and their standard deviations. For the standard deviation calculation, we assumed a correlation coefficient of 0.7 in cases lacking specific values, based on the expected strong correlation between initial and follow-up measurements.

A Q-Q plot led to the assumption of normal distribution for all variables. Mean score changes from baseline in each treatment group were compared using a random-effects model. Heterogeneity for each outcome was assessed using *I*^2^ statistic. Studies were considered statistically heterogenous when *p* < 0.1 and *I*^2^ > 50% (Higgins and Thompson, 2002).

Direct comparisons between the mean score change from the baseline of each treatment group to the follow-up period of interest were quantified through calculation of a mean difference and 95% confidence interval (CI). Mean differences were deemed statistically significant when *p* < 0.05.

## Results

**Figure 1.**
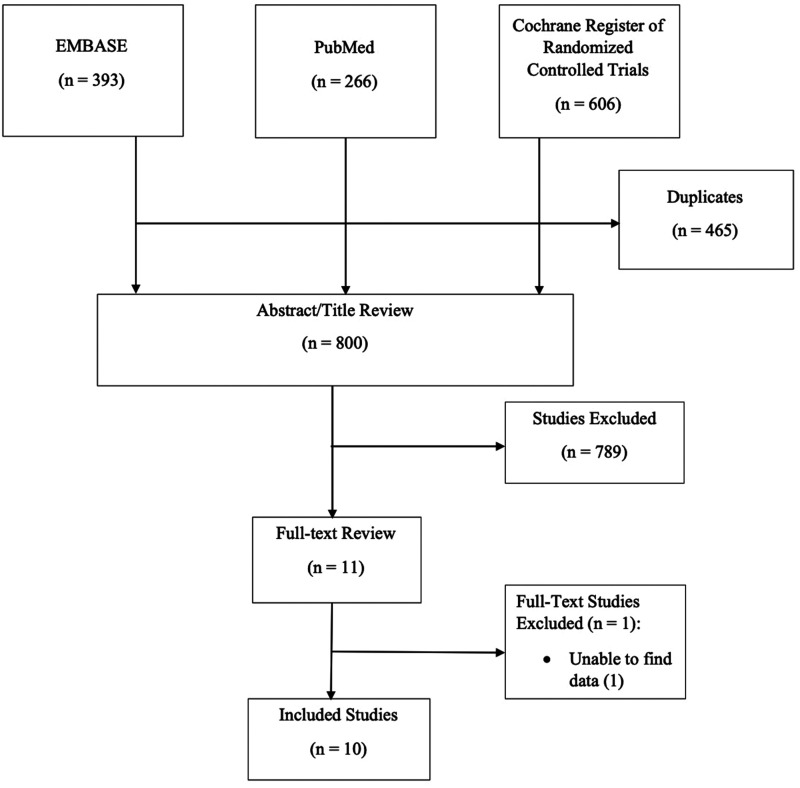
Flow diagram displaying the study selection process.

**Figure 2.**
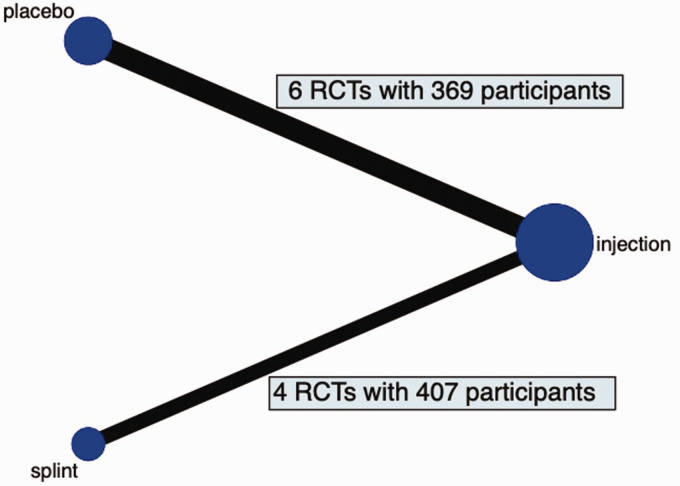
Network map of the included studies. Corticosteroid injections are included in all 10 studies and are directly compared to placebo injections (*n* = 6) and wrist splints (*n* = 4).

Our search identified 1265 articles: 393 from EMBASE; 266 from PubMed; and 606 from the Cochrane Register of Randomized Controlled Trials. Duplicates (*n* = 465) were omitted, leaving 800 studies. After screening the titles and abstracts, 789 studies were omitted for not meeting our selection criteria. A total of 11 full texts were analysed, and 10 studies were included into our network meta-analysis (Figures 1 and 2).

### Difference in symptom intensity after corticosteroid injection, placebo injection and splinting

There was no difference in FSS score changes between corticosteroid injection and splint or placebo injection at any time point. There was also no difference in pain intensity score changes between corticosteroid injection and placebo injection at any time point. At less than 3 months, people experienced a slightly greater decrease in SSS with corticosteroid injections compared to both splints and placebo injections, but this difference was smaller than the MCID. There was no difference in the change in SSS score change at 6 months.

Only one study compared the effects of corticosteroid injections and splints on pain intensity at 6 months and found that splints resulted in a greater decrease from baseline. There was no difference in pain intensity before 3 months (Tables 2 [Table table3-17531934241240380]–4).

**Table 2.** Network meta-analysis comparing splint, injection and placebo with injections serving as reference.

Outcome	No. of participants			Mean difference (95% CI)	Effect *p-*value	Heterogeneity (*I*^2^)	Heterogeneity *p*-value	No. of studies
	Injection	Placebo	Splint	Steroid injection (reference) vs. splint				
FSS <3 months	346	144	191	0.2 (−0.0 to 0.4)	0.11	62%	0.07	3
FSS at 6 months	242	73	166	0.7 (−0.7 to 2.2)	0.31	98%	**<0.01**	2
SSS <3 months	395	179	202	0.4 (0.2 to 0.7)	**<0.01**	73%	**0.01**	4
SSS at 6 months	279	108	166	0.5 (−0.7 to 1.8)	0.4	98%	**<0.01**	2
VAS <3 months	202	73	129	−0.1 (−1.9 to 2.1)	0.93	91%	**<0.01**	2
VAS at 6 months	190	73	118	−0.6 (−1.1 to −0.1)	**0.02**	N/A		1
DML <3 months	129	111	11	−0.3 (−0.9 to 0.3)	0.33	N/A		1
DML at 6 months	74	73	0					0
DSL <3 months	82	65	11	−0.6 (−1.1 to −0.1)	**0.03**	N/A		1
SNCV <3 months	74	73	0					0
SNCV at 6 months	74	73	0					0

Values in bold indicate a significant *p*-value.

CI: confidence interval; DML: distal motor latency; DSL: distal sensory latency; FSS: functional status scale; N/A: not applicable; SNCV: sensory nerve conduction velocity; SSS: symptom severity scale; VAS: visual analogue scale.

**Table 3. table3-17531934241240380:** Network meta-analysis comparing splint, injection and placebo with injections serving as reference.

Outcome	Steroid (reference) vs. placebo injection	Effect *p-*value	Heterogeneity (*I*^2^)	Heterogeneity *p*-value	No. of studies
FSS <3 months	0.3 (−0.0 to 0.7)	**0.08**	83%	**<0.01**	5
FSS at 6 months	−0.3 (−0.8 to 0.3)	0.31	87%	**<0.01**	3
SSS <3 months	0.4 (0.1 to 0.7)	**0.02**	85%	**<0.01**	6
SSS at 6 months	−0.2 (−0.7 to 0.3)	0.38	89%	**<0.01**	4
VAS <3 months	0.6 (−0.6 to 1.8)	0.3	87%	**<0.01**	3
VAS at 6 months	−0.5 (−2.7 to 1.7)	0.67	97%	**<0.01**	3
DML <3 months	0.2 (0.0 to 0.4)	**0.02**	28%	0.31	4
DML at 6 months	0.2 (−0.1 to 0.5)	0.14	0%	0.72	3
DSL <3 months	0.1 (−0.2 to 0.3)	0.55	42%	0.19	2
SNCV <3 months	−1.6 (−4.3 to 1.1)	0.25	73%	**0.03**	3
SNCV at 6 months	−0.2 (−3.4 to 2.9)	0.89	78%	**0.01**	3

Values in bold indicate a significant *p*-value.

DML: distal motor latency; DSL: distal sensory latency; FSS: functional status scale; SNCV: sensory nerve conduction velocity; SSS: symptom severity scale; VAS: visual analogue scale.

**Table 4.** Mean difference (95% CI) with steroid injection as the reference treatment

Study	Participants per modality			Outcomes			
	Injection	Placebo	Splint	FSS <3 months	FSS at 6 months	SSS <3 months	SSS at 6 months
De Moraes et al. (2021)	52	0	48	0.3 (0.0 to 0.6)	1.5 (1.2 to 1.8)	0.8 (0.5 to 1.0)	1.2 (0.9 to 1.4)
Chesterton et al. (2018)	116	0	118	0.3 (0.1 to 0.4)	0.0 (−0.2 to 0.2)	0.4 (0.2 to 0.5)	−0.1 (−0.3 to 0.1)
So et al. (2018)	25	0	25	0.0 (−0.2 to 0.2)		0.3 (−0.0 to 0.6)	
Celiker et al. (2002)	12	0	11			0.1 (−0.5 to 0.7)	
Wu et al. (2018)	27	27	0	−0.3 (−0.5 to 0.0)	−0.8 (−1.0 to −0.5)	−0.3 (−0.6 to −0.0)	−0.9 (−1.1 to −0.6)
Karadaş et al. (2012)	20	19	0	0.1 (−0.3 to 0.5)	0.11 (−0.3 to 0.5)	0.3 (−0.0 to 0.6)	0.2 (−0.1 to 0.5)
Peters-Veluthamaningal et al. (2010)	36	33	0	0.7 (0.4 to 1.1)		0.6 (0.3 to 0.9)	
Babaei-Ghazani et al. (2022)	27	27	0	0.5 (0.1 to 0.9)	−0.1(−0.5 to 0.3)	0.4 (0.1 to 0.7)	0.1 (−0.3 to 0.5)
Armstrong et al. (2004)	43	38	0	0.5 (0.2 to 0.8)		0.6 (0.3 to 0.9)	
Atroshi et al. (2013)	37	35	0			0.9 (0.5 to 1.3)	−0.3 (−0.7 to 0.1)

CI: confidence interval; FSS: functional status scale; SSS: symptom severity scale.

### Difference in nerve conduction study measures after corticosteroid injection, placebo injection and splinting

One study compared the effects of corticosteroid injections and splinting on distal motor latency (DML) and distal sensory latency (DSL) at less than 3 months and found no difference in DML and a greater reduction in DSL from baseline with wrist splinting.

Two studies compared the effects of corticosteroid injections and placebo on DSL and found no difference at less than 3 months. In addition, no differences were observed between corticosteroid and placebo injections for DML at 6 months and sensory nerve conduction velocity at both less than 3 months and 6 months (Tables 2, 3 and 5). At less than 3 months, the score change in DML was slightly greater after corticosteroid injection compared to placebo injection.

**Table 5.** Mean difference (95% CI) with steroid injection as the reference treatment

Study	Outcomes						
	VAS <3 months	VAS at 6 months	DML <3 months	DML at 6 months	DSL <3 months	SNCV <3 months	SNCV at 6 months
De Moraes et al. (2021)							
Chesterton et al. (2018)	1.1 (0.5 to 1.6)	−0.6 (−1.1 to −0.1)					
So et al. (2018)							
Celiker et al. (2002)	−1 (−2.1 to 0.1)		−0.3 (−0.9 to 0.3)		−0.6 (−1.1 to −0.1)		
Wu et al. (2018)	−0.1 (−0.8 to 0.6)	−2.6 (−3.3 to −1.9)	0.2 (−0.4 to 0.8)	0 (−0.6 to 0.6)		−0.1 (−2.8 to 2.6)	1.4 (−1.3 to 4.1)
Karadaş et al. (2012)	1.8 (1.2 to 2.3)	1.2 (0.8 to 1.6)	0.6 (0.1 to 1.1)	0.2 (−0.2 to 0.7)		−4.5 (−7.1 to −1.8)	−3.5 (−6.1 to −0.9)
Peters-Veluthamaningal et al. (2010)							
Babaei-Ghazani et al. (2022)	0.1 (−0.9 to 1.1)	−0.1 (−1.2 to 0.9)	0.3 (−0.2 to 0.7)	0.3 (−0.1 to 0.8)	−0.2 (−0.6 to 0.3)	−0.33 (−4.3 to 1.1)	1.4 (−1.1 to 3.8)
Armstrong et al. (2004)			0.2 (0.1 to 0.2)		0.2 (0.1 to 0.3)		
Atroshi et al. (2013)							

CI: confidence interval; DML: distal motor latency; DSL: distal sensory latency; SNCV: sensory nerve conduction velocity; VAS: visual analogue scale.

## Discussion

Based on a single RCT, the treatment guidelines of the AAOS indicate that there is strong evidence suggesting that methylprednisolone steroid injection improves patient-reported outcomes (Graham et al., 2016). However, they did not address the ability of corticosteroid injections to modify the natural course of the median neuropathy. In our network meta-analysis on CTS (symptom palliation) and idiopathic median neuropathy at the carpal tunnel (disease modification), we found that that corticosteroid injection modestly reduced distal motor latency for less than 6 months without altering DSL or sensory nerve conduction velocity. This small transient change in pathophysiology does not translate to clinically relevant palliation of symptoms compared to placebo. Patients should be aware that most improvement after injection may be due to non-specific effects, and injections do not lead to lasting pathophysiological changes.

The observations that corticosteroid injections had a modest benefit in SSS, which is below the MCID to splinting and placebo at less than 3 months, no benefit at 6 months and no benefit in FSS, suggest that a clinically relevant palliation from corticosteroid injection is unlikely. These findings are consistent with previous systematic reviews indicating that corticosteroid injections produce a statistically significant but not clinically meaningful improvement in CTS symptom severity compared to placebo injections within 3 months of injection and no difference later (Ashworth et al., 2023; Karjalanen et al., 2022).

In contrast, a network meta-analysis comparing the efficacy of different corticosteroid-injection approaches and a placebo injection in treating CTS found that corticosteroid injections generally outperformed placebo in reducing symptom intensity within the first 3 months, regardless of the approach (Chen et al., 2015). Those authors included the studies by Armstrong et al. (2004), Atroshi et al. (2013), Karadaş et al. (2012) and Peters-Veluthamaningal et al. (2010) as we did, but they also included six studies that we excluded because those studies did not compare corticosteroid injections to other treatment options. Notably, Chen et al. (2015) did not compare the magnitude of difference in the reported outcomes to the MCID.

The 2016 AAOS Clinical Practice Guidelines based their conclusion on the study by Atroshi et al. (2013), which compared the BCTQ symptom severity scores between 40 mg and 80 mg corticosteroid dosages to a placebo injection. This study, included in our meta-analysis, showed significant SSS differences at 5 and 10 weeks for both dosages versus placebo, but they did not exceed the MCID. De Kleermaeker et al. (2019) identified a relative MCID of 1.38 for BCTQ symptom severity scores among patients with average baseline scores of three. Neither intervention achieved this MCID at the 5- and 10-week follow-ups (Atroshi et al., 2013, De Kleermaeker et al., 2019). The lack of clinically relevant symptom palliation from actual corticosteroid injections compared to simulated (placebo) injections highlights the relevance of non-specific treatment effects, which are often more pronounced in invasive treatments. Considering patient preferences against treatments not exceeding non-specific effects and the desire for informed choices, it is possible that patients, when informed of these findings, might decline an offer of corticosteroid injection (Bandell et al., 2020). There may be an ethical duty to limit false hope associated with misunderstanding the injection as potentially disease modifying and also distraction from making a long-term plan if the corticosteroid injection provides transient limited palliation (Kaile and Bland, 2018).

The findings of a slightly lower DML at 3 months after corticosteroid injection versus placebo, better DSL within 3 months compared to splints, no other electrodiagnostic differences and no DML or DSL changes at 6 months suggest that injections may only offer minor, temporary reduction of median neuropathy, with no lasting disease-modifying effect. The limited sample size of 129 patients across five studies indicates that the effect of corticosteroid injections on pathophysiology is not well studied. A larger, randomized, placebo-controlled trial focusing on median neuropathy severity would help determine if corticosteroid injections can meaningfully modify the natural history of idiopathic median neuropathy at the carpal tunnel.

The present study has some limitations. First, the inclusion of 776 patients and 819 hands challenges the assumption of independence of our statistical tests (Park et al., 2010). Despite this, acquiring complete individual data in systematic reviews is often challenging, and only a minor fraction (5.3%, 43/819) had both hands included (Teunis et al., 2015). Second, potential performance bias arose from inadequate blinding in eight studies, particularly in comparisons involving corticosteroid injections and splinting. While patient blinding was feasible in placebo-controlled scenarios, it was not possible in splinting comparisons. This could favour corticosteroid injections, yet our findings suggest minimal or no benefit. Third, there is no consensus reference standard for the diagnosis of CTS, and inclusions varied. While this is likely an accurate representation of clinical practice, it is important to consider that broad diagnostic parameters might encompass individuals without idiopathic median neuropathy at the carpal tunnel, potentially confounding the assessment of corticosteroid injections’ efficacy on median neuropathy. Fourth, we only assessed a single corticosteroid injection. Given the outcomes with a single injection, it is unlikely that multiple injections could significantly impact palliation or modify disease. Fifth, our comparison of electrodiagnostic measures after splinting included only 11 patients, so we cannot draw any firm conclusions about the effect of splinting on median neuropathy severity. Sixth, we assumed standard deviations for mean score changes, based on a presumed strong correlation between baseline and follow-up scores. However, actual standard deviations might significantly differ from our reported values. Seventh, there is a possibility that not all relevant studies were included as our search was limited to those published in English. However, we did not find additional studies in the references of the included studies. Finally, this study was limited in sufficiently capturing the distinction between corticosteroid injections and alternative treatment modalities in relation to DSL. With a maximum of two studies per treatment modality examining this discrepancy, drawing definitive conclusions about their impact on DSL is difficult.

There is a risk that a patient who experiences palliation after a corticosteroid injection might misinterpret this as a cure, while the disease can continue to progress, potentially resulting in permanent neuropathy.

## Supplemental Material

sj-pdf-1-jhs-10.1177_17531934241240380 - Supplemental material for Steroid versus placebo injections and wrist splints in patients with carpal tunnel syndrome: a systematic review and network meta-analysis Supplemental material, sj-pdf-1-jhs-10.1177_17531934241240380 for Steroid versus placebo injections and wrist splints in patients with carpal tunnel syndrome: a systematic review and network meta-analysis by Ebubechi Adindu, Sina Ramtin, Ali Azarpay, David Ring and Teun Teunis in Journal of Hand Surgery (European Volume)

sj-pdf-2-jhs-10.1177_17531934241240380 - Supplemental material for Steroid versus placebo injections and wrist splints in patients with carpal tunnel syndrome: a systematic review and network meta-analysis Supplemental material, sj-pdf-2-jhs-10.1177_17531934241240380 for Steroid versus placebo injections and wrist splints in patients with carpal tunnel syndrome: a systematic review and network meta-analysis by Ebubechi Adindu, Sina Ramtin, Ali Azarpay, David Ring and Teun Teunis in Journal of Hand Surgery (European Volume)

sj-pdf-3-jhs-10.1177_17531934241240380 - Supplemental material for Steroid versus placebo injections and wrist splints in patients with carpal tunnel syndrome: a systematic review and network meta-analysis Supplemental material, sj-pdf-3-jhs-10.1177_17531934241240380 for Steroid versus placebo injections and wrist splints in patients with carpal tunnel syndrome: a systematic review and network meta-analysis by Ebubechi Adindu, Sina Ramtin, Ali Azarpay, David Ring and Teun Teunis in Journal of Hand Surgery (European Volume)

sj-pdf-4-jhs-10.1177_17531934241240380 - Supplemental material for Steroid versus placebo injections and wrist splints in patients with carpal tunnel syndrome: a systematic review and network meta-analysis Supplemental material, sj-pdf-4-jhs-10.1177_17531934241240380 for Steroid versus placebo injections and wrist splints in patients with carpal tunnel syndrome: a systematic review and network meta-analysis by Ebubechi Adindu, Sina Ramtin, Ali Azarpay, David Ring and Teun Teunis in Journal of Hand Surgery (European Volume)
