# Supplementary material for: Steroid versus placebo injections and wrist splints in patients with carpal tunnel syndrome: a systematic review and network meta-analysis
Source: J Hand Surg Eur Vol. 2024 Mar 28;49(10):1209–17. doi: 10.1177/17531934241240380 (PMC11523550; doi:10.1177/17531934241240380)
Supplement: sj-pdf-1-jhs-10.1177_17531934241240380 - Supplemental material for Steroid versus placebo injections and wrist splints in patients with carpal tunnel syndrome: a systematic review and network meta-analysis [file sj-pdf-1-jhs-10.1177_17531934241240380.pdf]

Appendix A

Search Strategy

(“median neuropathy” OR “carpal tunnel syndrome” OR CTS OR “median nerve compression” OR “median nerve entrapment”) AND (glucocorticoids OR *steroid* OR triamcinolone OR methylprednisolone OR orthosis OR splint OR injection OR PRP OR “platelet-rich plasma”)
