# Supplementary material for: Steroid versus placebo injections and wrist splints in patients with carpal tunnel syndrome: a systematic review and network meta-analysis
Source: J Hand Surg Eur Vol. 2024 Mar 28;49(10):1209–17. doi: 10.1177/17531934241240380 (PMC11523550; doi:10.1177/17531934241240380)
Supplement: sj-pdf-2-jhs-10.1177_17531934241240380 - Supplemental material for Steroid versus placebo injections and wrist splints in patients with carpal tunnel syndrome: a systematic review and network meta-analysis [file sj-pdf-2-jhs-10.1177_17531934241240380.pdf]

Risk of bias domains

Study	D1	D2	D3	D4	D5	Overall
de Moraes, et al. 2021	+	-	+	+	+	-
Chesterton, et al. 2018	+	-	+	+	+	-
Wu, et al. 2018	+	-	+	+	+	-
So, et al. 2017	+	-	+	+	+	-
Karadaş, et al. 2012	+	-	X	+	+	X
Peters-Veluthamaningal, et al. 2010	+	-	+	+	+	-
Celiker, et al. 2002	+	-	+	+	+	-
Babaei-Ghazani, et al. 2022	+	-	+	+	+	-
Armstrong, et al. 2004	+	+	+	+	+	+
Atroshi, et al. 2013	+	+	+	+	+	+

Domains:
D1: Bias arising from the randomization process.
D2: Bias due to deviations from intended intervention.
D3: Bias due to missing outcome data.
D4: Bias in measurement of the outcome.
D5: Bias in selection of the reported result.

Judgement
X High
- Some concerns
+ Low
