# Supplementary figures and images for: Steroid versus placebo injections and wrist splints in patients with carpal tunnel syndrome: a systematic review and network meta-analysis
Source: J Hand Surg Eur Vol. 2024 Mar 28;49(10):1209–17. doi: 10.1177/17531934241240380 (PMC11523550; doi:10.1177/17531934241240380)

## Appendix B

### Publication Bias Funnel Plots

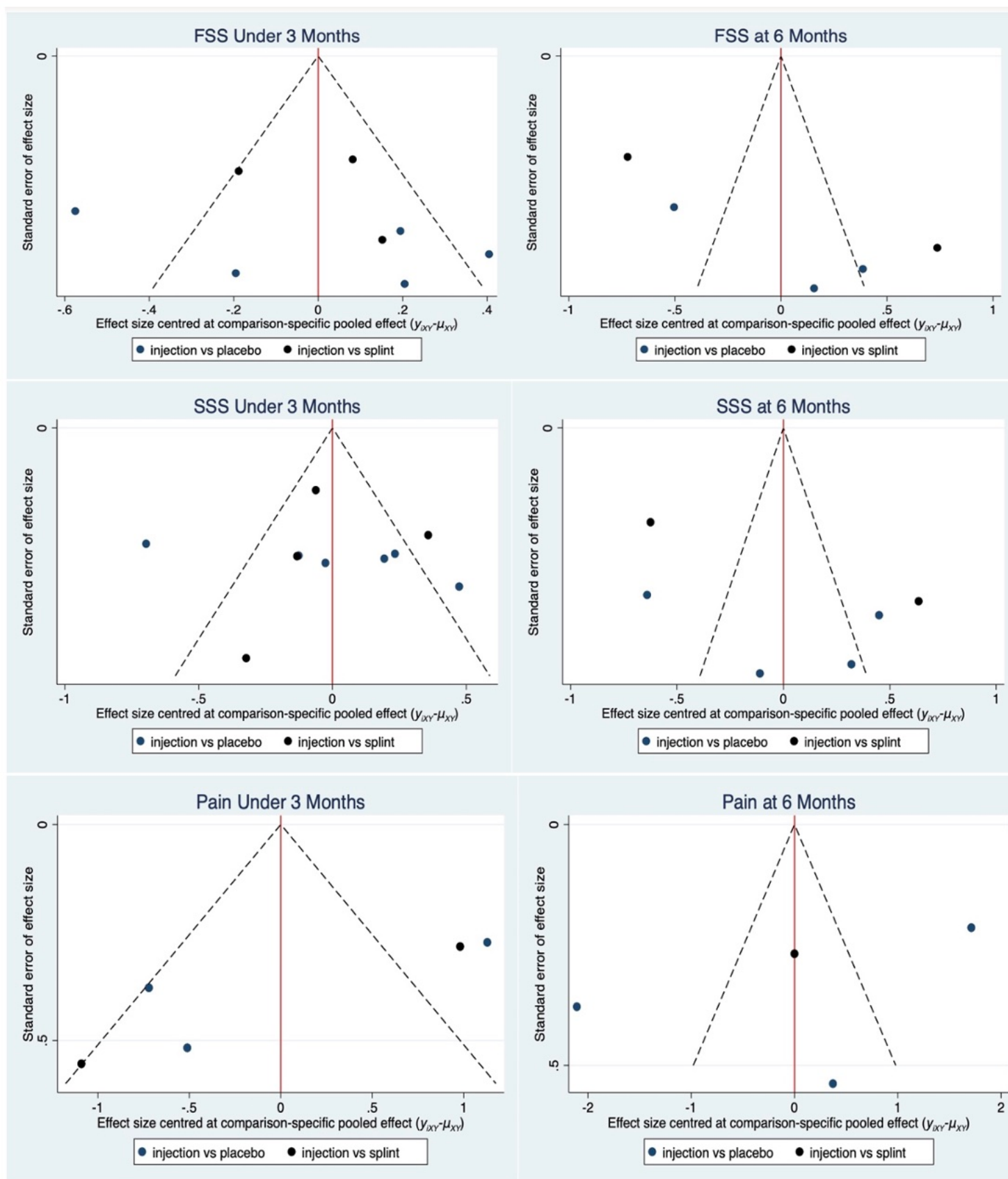

Supplement: sj-pdf-3-jhs-10.1177_17531934241240380 - Supplemental material for Steroid versus placebo injections and wrist splints in patients with carpal tunnel syndrome: a systematic review and network meta-analysis [file sj-pdf-3-jhs-10.1177_17531934241240380.pdf]

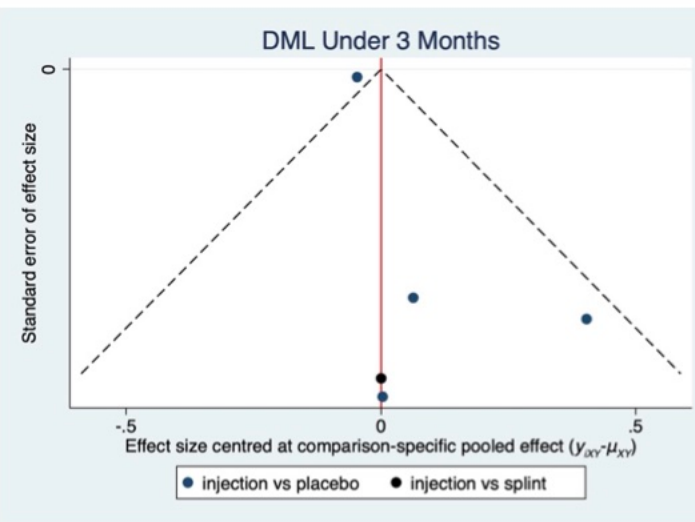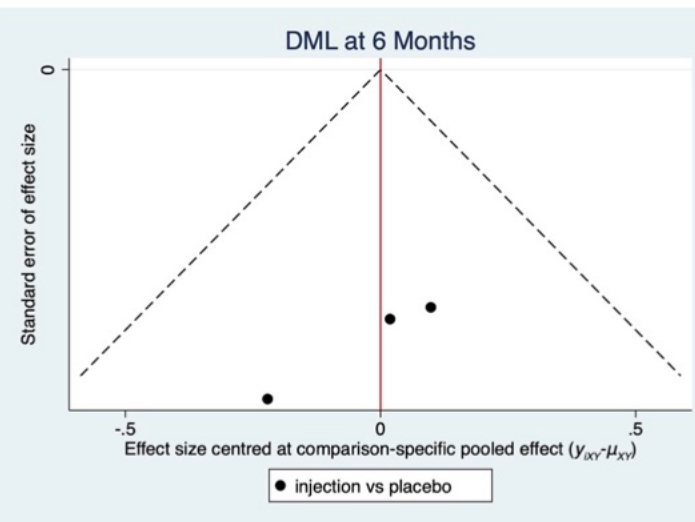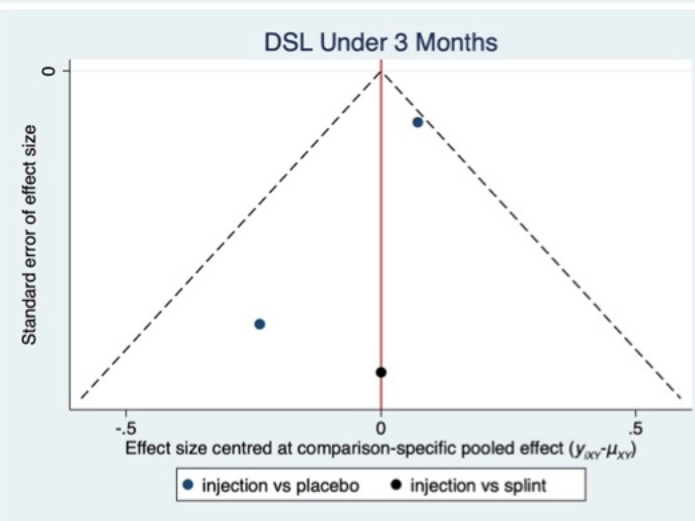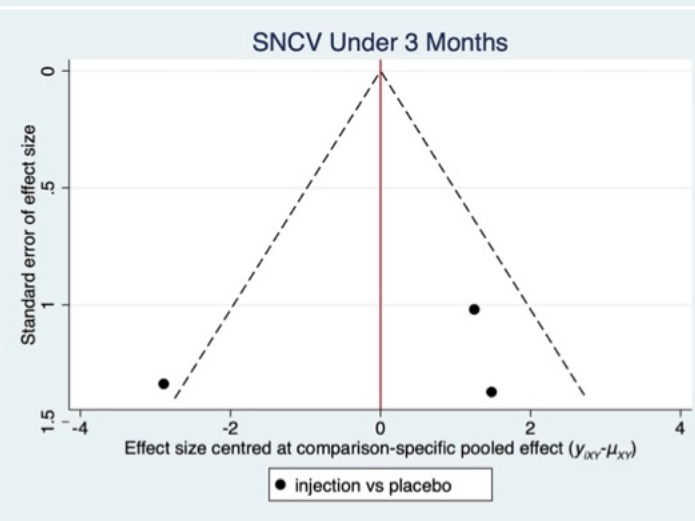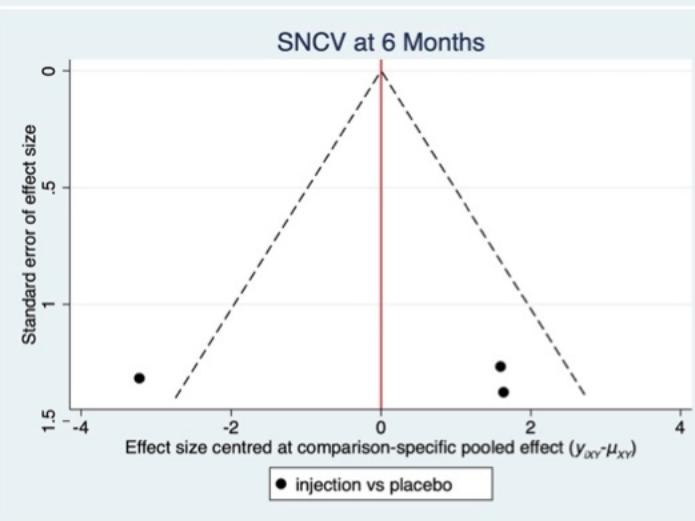

Supplement: sj-pdf-4-jhs-10.1177_17531934241240380 - Supplemental material for Steroid versus placebo injections and wrist splints in patients with carpal tunnel syndrome: a systematic review and network meta-analysis [file sj-pdf-4-jhs-10.1177_17531934241240380.pdf]
